# Bile acids at the cross-roads of gut microbiome–host cardiometabolic interactions

**DOI:** 10.1186/s13098-017-0299-9

**Published:** 2017-12-28

**Authors:** Paul M. Ryan, Catherine Stanton, Noel M. Caplice

**Affiliations:** 1 APC Microbiome Institute, Biosciences Institute, University College Cork, Cork, Ireland; 2 Centre for Research in Vascular Biology, University College Cork, Co. Cork, Ireland; 3 Food Biosciences Department, Teagasc Food Research Centre, Moorepark, Fermoy, Co. Cork, Ireland

**Keywords:** Microbiome, Bile acids, Cardiovascular disease, Metabolic syndrome

## Abstract

While basic and clinical research over the last several decades has recognized a number of modifiable risk factors associated with cardiometabolic disease progression, additional and alternative biological perspectives may offer novel targets for prevention and treatment of this disease set. There is mounting preclinical and emerging clinical evidence indicating that the mass of metabolically diverse microorganisms which inhabit the human gastrointestinal tract may be implicated in initiation and modulation of cardiovascular and metabolic disease outcomes. The following review will discuss this gut microbiome–host metabolism axis and address newly proposed bile-mediated signaling pathways through which dysregulation of this homeostatic axis may influence host cardiovascular risk. With a central focus on the major nuclear and membrane-bound bile acid receptor ligands, we aim to review the putative impact of microbial bile acid modification on several major phenotypes of metabolic syndrome, from obesity to heart failure. Finally, attempting to synthesize several separate but complementary hypotheses, we will review current directions in preclinical and clinical investigation in this evolving field.

## Background

The human metagenome consists of the host genome and a symbiont microbiome of over 10 trillion bacteria predominantly located within the host gastrointestinal tract. Both genomes have co-evolved over millennia with the microbiome expressing a genetic diversity greater than that of the host [1]. The microbiome mass has been estimated at ~ 4 × 10^13^ [1], with distribution within the intestine highly segmented between the upper/small intestine and the large intestine [2]. Such segmentation of the predominant bacterial mass within the large intestine is likely a result of competitive evolutionary forces that have ensured the host has ‘first shot’ at dietary substrates. Interaction of the host/microbiome with the external environment through the ingestion of food thus plays a major role in determining the beneficial or detrimental effects of nature on human health [3]. In fact, during the post industrial age, the human microbiome has evolved to a much greater extent than the previous 4 million years of hominid existence [4]. Thus, we stand at the threshold of a new era of discovery where fundamental insights into the nature of biologic interaction of our “other genome” with our own host physiology will illuminate new mechanisms of understanding of the role of environment in diseases of modern man. Nowhere is this more relevant than the cardiovascular system which is afflicted by a spectrum of diseases that have become the scourge of our increasingly narrow, high-calorie, high-sugar and fat-rich ‘Western’ diet.

Seminal studies by Hazen and co-workers have already neatly highlighted the deleterious interaction between phosphatidylcholine in the human diet and expansion of selected gut bacteria that produce trimethylamine, which is further metabolized to trimethylamine-*N*-oxide, a metabolite that correlates with increased risk of cardiovascular disease (CVD; [5–8]). Despite the compelling preclinical and clinical evidence now available, a recently published meta-analysis of 18,076 incident CVD events, 5343 CVD deaths and 184,010 subjects revealed no significant link between quaternary amine consumption and hard CVD outcomes (i.e. incidence and mortality) [9]. With this in mind, and in the light of several recent extensive reviews on the trimethylamine-*N*-oxide pathway, this aspect of the microbiome–cardiometabolic interaction will not be discussed further herein.

In this review, we intend to address another complementary aspect of the gut microbiome–host metabolism interaction, which is modulated centrally by microbial-mediated bile acid modification. Herein, we examine the relationship of the gut microbiome to disease entities ranging from obesity, hyperlipidemia, insulin resistance and hypertension (i.e. metabolic syndrome) through heart failure with preserved ejection fraction to atherosclerotic disease and complications of stroke, myocardial infarction and peripheral vascular disease. This review pulls from the modest cohort of randomized controlled trials currently available, which is supported by preclinical mechanistic data and clinical observational studies of bariatric surgery patients. We aim to put into context the most current knowledge on how the microbiome, through bile acid manipulation, has the potential to impact on host cardiovascular and metabolic health in a manner which outlines not only pathways, but also the potential environmental aspects that contribute to this tipping-point and potential novel therapies that might derive from it.

## Microbial bile modification

Bile acids have systemic applications that far surpass their mechanical role as intestinal detergents. The de novo synthesis of bile acids from cholesterol occurs in hepatocytes through two pathways, termed classical and alternative, which are facilitated by cytochrome P enzymes CYP7A1 and CYP27A1, respectively. In a homeostatic state, bile acids are recycled 4–12 times per day, with less than 5% of secreted bile acids being excreted. However, bile modification by members of the gut microbiome can greatly influence the efficacy at which these compounds are reabsorbed and recycled. Naïve conjugated bile salts, secreted into the descending duodenum, undergo several microbial modification reactions, the first of which involves deconjugation to primary bile acids by bile salt hydrolase (BSH) enzymes. This process involves the removal of an amine group, either taurine or glycine, from the conjugated bile salt. Bile salts can be highly cytotoxic to microorganisms, these enzymes therefore likely evolved as a detoxification mechanism and as such, are expressed in abundance by the major microbial phyla found in the human gastrointestinal tract [10]. This reaction ‘unlocks’ the sterol molecule to a plethora of downstream microbial enzymes, including 7α-dehydroxylase [11]. The detailed enzymatic processes of these reactions have been neatly reviewed and presented previously by Martin and colleagues [12]. Intriguingly, microbial enzymes have the potential to create dozens of different bile acid-derived molecules [13, 14], each with varying capacity to function as signaling molecules within the host system. In addition, these modifications alter the reabsorptive capacity of bile acids leading to obligate excretion. Due to this central role of the gut microbiome in profoundly shaping the whole bile pool and the diversity of its profile, bile acids offer a convincing pathway of microbe–host crosstalk in the context of overall host metabolism. In a clinical setting, bile acids may represent potential biomarkers of metabolic health and disease which are highly trackable through analytic techniques [15], while also providing several potentially potent pathways that may be targeted in the pursuit of novel therapeutics.

## Bile modulates host metabolic function

The gut microbiome potentially represents a “bounty or burden” in terms of its metabolic capacity and how this impacts upon host cardiometabolic health. In line with this, the variety of microbes that humans host appear to have influence over virtually every metabolic process; altering adiposity, liver lipid metabolism and glucose metabolism, as well as heart and vascular function [16]. Current research in the area of microbiome–host crosstalk implicates a number of familiar metabolic groups in this interdependency—including endotoxins, short-chain fatty acids, several neuroactive peptides and microbially-modified bile acids, amongst others. The significance of bile acids, of intestinal and hepatic origin, as paracrine and endocrine signaling molecules with systemic reach has only recently been highlighted [17]. These molecules impact significantly on pathways intimately involved in inflammation, host lipid, cholesterol and glucose metabolism, while also demonstrating the potential to alter circadian rhythm [18] and immunity [19], in preclinical models. We now address some of the compelling preclinical and clinical data detailing these intricate interactions (Table 1).

**Table 1:** Bile acid receptors, ligands and cardiometabolic physiological effects

Receptor	Natural and synthetic ligands	Serum concentration (nM) [107]	Relative potency	Reported physiological effects				
				Adiposity	glucose metabolism	Lipid metabolism	Vascular disease	HF and HTN
FXR	CDCA	380		↑ BAT [39]	↓ Glucose ↓ Insulin [147]	↓ TAG [147]	↑ RCT [86]	↓ BP [98]
	CA	440						
	DCA	320						
	LCA	< LOD						
	UDCA	43						
	WAY-362450	N/A		–	–	↓ TAG [148] ↓ Cholesterol [89, 149]	↓ Atherosclerosis [88, 89]	–
	GW4064	N/A		–	↑ Glycogen [150] ↑ Insulin sensitivity [151] ↓ Glucose	↓ TAG/FFA ↓ Cholesterol [152–154]	↑ RCT [87]	–
	OCA	N/A		↑ BAT ↓ WAT [113]	↓ Glucose [113]	↑ LDL [113] ↓ HDL [117]	↑ RCT [155]	↓ BP [78]
	Fexaramine	N/A		↑ BAT [52]	↓ Glucose ↓ Gluconeogenesis ↓ Insulin [52]	↓ Cholesterol [52]	–	–
TGR5	LCA	< LOD		↑ BAT ↑ T4 ⟶ T3 [40]	↓ Glucose ↓ Insulin [40]	–	↑ NO ↓ Monocyte infiltration [94]	–
	TLCA	0.33						
	CA	440						
	DCA	320						
	CDCA	380						
	6-EMCA/INT-777	N/A		↑ BAT ↓ WAT [156]	↑ GLP-1 [156]	↓ TAG/NEFA [156]	↓ Atherosclerosis ↓ Foam cell [93]	–

*BAT* brown adipose tissue, *WAT* white adipose tissue, *T4* thyroxine, *T3* triiodothyronine, *GLP-1* glucagon-like peptide-1, *TAG* triacylglycerides, *FFA* free fatty acids, *NEFA* non-esterified fatty acids, *RCT* reverse cholesterol transport, *NO* nitric oxide, *BP* blood pressure, *LOD* limit of detection, *N/A* not applicable

### Weight management and obesity

Obesity represents a central facet of the metabolic syndrome and a preponderance of microbiome–host metabolism interaction studies have focused on it. One of the most important early clinical discoveries was that perturbations in the gut microbiome caused by antibiotic administration during early development can have lasting effects on both the composition and richness of the microbiome [20], with deleterious consequences for lifelong metabolic health of the host [21–24]. Microbiome richness, generally regarded as a proxy for its metabolic capabilities or functionality, has in turn been shown to correlate tightly with human inflammatory and metabolic markers [25].

Some of the most convincing clinical data to indicate a role of the gut microbiome in obesity has arisen from bariatric surgery studies. The mechanism underlying efficacy of these obesity-curbing surgeries, including vertical sleeve gastrectomy and Roux-en Y gastric bypass, has long puzzled researchers. Observational clinical studies have catalogued the gut microbiome alterations observed in these patients, consistently reporting increased Proteobacteria and decreased Bacteroides levels [26]. In addition, serum bile profiling reveals significant fluctuation post vertical sleeve gastrectomy, including increased glycoursodeoxycholic acid (GUDCA) coupled with decreased glycocholic acid (GCA), taurocholic acid (TCA) and deoxycholic acid (DCA) [27]. Ryan et al. were first to highlight the role of microbial bile acid modification in the beneficial effects of successful surgeries of this nature—i.e. those which led to significant weight/BMI reduction, improved lipid and glucose metabolism [28]. This group of investigators had previously demonstrated a positive relationship between satiety/weight-loss and expression of murine fibroblast growth factor (FGF) 15 (equivalent to FGF19 in Man). FGF15 is a factor tightly modulated by the bile acid nuclear receptor farnesoid X receptor (FXR) [29], which is a critical intracellular sensor regulating bile acid and cholesterol homeostasis [30]. This underscored a molecular rather than mechanical foundation for the efficacy of bariatric surgery, with bile acid signaling at the core [31]. Subsequent preclinical studies have further elaborated on this pathway [32–34], while several recent clinical trials have solidified the perceived role of bile acid-FXR-FGF19 signaling in obesity [35–37].

In terms of the weight attenuating effects observed in mice, several groups have now pointed to the potent endogenous FXR antagonist, TβMCA, as a key modulator of host adiposity. The Wallenberg Laboratory initially identified bile acid enrichment in the enterohepatic system of germ-free mice, demonstrating additionally the regulation of bile acid synthesis through the FXR-FGF15 axis [38]. The same group showed that microbiome-mediated depletion of FXR antagonists, such as TβMCA, is implicated in obesity induced by high-fat diet [34]. However, clinical relevance of these findings remains unclear, as TβMCA and related bile acids are not synthesised in the human system.

Intriguingly, bile acids have also shown the propensity to impact upon mammalian energy expenditure through the thyroid system. Watanabe and colleagues first demonstrated this effect in mice through application of the Takeda-G-protein-receptor 5 (TGR5) agonist, cholic acid (CA) [39]. The effects were deemed to be mediated by the induction of the cyclic-AMP-dependent thyroid hormone activating enzyme type 2 iodothyronine deiodinase, thereby increasing energy expenditure in brown adipose tissue (BAT). A small-scale randomized controlled trial crossover study recently further explored the impact of a 2-day chenodeoxycholic acid (CDCA) oral intervention on BAT activity and energy expenditure in 12 healthy females [40]. The results indicated a substantial increase in both primary outcome parameters, which were determined by positron emission tomography–computed tomography scan and serum thyroid hormones, respectively. The authors mined down to the relevant receptor activity through ex vivo human BAT culture experiments with CDCA and specific bile acid receptor agonists, in which the G-protein coupled receptor TGR5 was confirmed. TGR5 is one of several membrane-bound receptors highly expressed in enterocytes of the ileum and colon, which is readily activated by several naïve and microbially-modified bile acids. These studies indicate a potential application of TGR5-agonists as anti-obesogenic or indeed anti-diabetic pharmaceuticals, as BAT is known to stimulate insulin sensitivity [41].

### Insulin secretion and sensitivity

The gut microbiome has been proposed as a highly sensitive and specific tool for the identification of insulin insensitivity [42–44], although this is currently of limited clinical utility. The metabolic capacity of the gut microbiome appears to be fundamental to host glucose metabolism, with metabolites such as branched-chain amino acids [45] and succinate [46] most recently being reported as negatively and positively correlated with insulin sensitivity, respectively. Microbiome-mediated succinate production, for instance, was found to impact directly upon glucose homeostasis by inducing intestinal gluconeogenesis, while branched-chain amino acid production, in this case by *Prevotella copri* and *Bacteroides vulgatus*, has previously been associated with metabolic dysfunction and future insulin resistance [47, 48].

The potential association of bile acid profile with diabetic state has been acknowledged for decades [49], yet the consequences of this interaction appear to have remained relatively underappreciated. In addition to the role of bile-mediated FXR signaling in correcting obesity, there are indications that this pathway may also impact insulin sensitivity [50]. A prospective study of diabetic women undergoing bariatric surgery identified a significant increase in circulating FGF19 following the procedure, which appeared to contribute to mitochondrial recovery and insulin sensitivity [51]. Moreover, targeted agonism of enterocyte FXR receptors with Fexaramine has previously been shown to trigger thermogenesis, adipocyte browning and improved insulin sensitivity [52]. Indeed, there is indication now that FGF19 may acutely reduce circulating glucose levels through suppression of the hypothalamus–pituitary–adrenal axis [53].

Interestingly, TGR5 has been shown to modulate secretion of the incretin GLP-1 [54], a gut hormone which has a range of beneficial systemic effects including satiation, stimulation of insulin secretion and sensitivity, glucogenesis and lipolysis [55]. Moreover, TGR5 has been implicated in the improved glucose homeostasis already attributed to bariatric surgery [56]. While GLP-1 has also been shown to have cardioprotective effects, in terms of inhibiting triglyceride-rich chylomicron formation [57, 58], attenuation of atherogenic plaque inflammation [59] and modulation of cardiomyocyte function [60, 61], the importance of bile acid-mediated GLP-1 secretion in the context of CVD has not yet been fully explored. Until this point, we have discussed FXR and TGR5 bile signaling in isolation of each other; however, we now have clear evidence that FXR also stimulates GLP-1 secretion through TGR5 upregulation, thereby ameliorating lipid metabolism in mice [62].

### Hepatic lipid metabolism

There is an established link between the composition and functionality of the gut microbiome and host lipid metabolism [63, 64]. A recent investigation into this relationship probed the 893-subject LifeLines-DEEP cohort dataset in an attempt to correlate microbial taxa relative abundances with metabolic markers such as BMI, cholesterol and triglycerides [65]. The study reported a range of correlations, with the microbiome explaining ~ 5% of BMI, triglyceride and HDL-C variance independent of confounding factors, such as age, sex and genetic risk factors. Interestingly, of those most inversely correlated with BMI and circulating triglyceride levels were bile acid metabolizing taxa *Bacteroidales* and *Clostridiaceae*.

At the center of microbial bile metabolism are the BSH enzymes, which deconjugate bile salts, thereby rendering them exposed to numerous additional modifications. Joyce et al. previously demonstrated the ability of a recombinant BSH-expressing microbe to substantially alter the host circulating bile acid profile and, in turn, markers of host lipid metabolism and circadian rhythm [66]. In support of this concept, a series of relatively small clinical studies have demonstrated a role for BSH-expressing microbes in the treatment of mildly hypercholesterolaemic individuals. The bile-modifying microbe, *Lactobacillus reuteri* NCIMB 30242, has repeatedly been shown to modestly reduce circulating non-HDL-C levels, as well as other inflammatory markers of atherogenesis [67, 68]. Furthermore, it has been reported that the BSH-active probiotic cocktail VSL#3, which has been demonstrated to induce bile acid synthesis through the aforementioned FXR-FGF15 axis [69], can beneficially alter serum cholesterol and the lipid profile of critically ill patients [70]. This same bile-modifying therapeutic has also been shown to significantly reduce the severity of non-alcoholic fatty liver disease (NAFLD) in obese children [71].

From a clinical standpoint, clean receptor agonists/antagonists would be more attractive than enzyme-secreting microbes, in terms of ease of use, efficacy and safety. In line with this, several bile acid receptor agonists/antagonists have been developed in recent years in an attempt to target this microbial–host crosstalk in certain metabolic pathologies [72]. FXR has been shown, through murine knock-out models, to play a central role in cholesterol homeostasis [73] and is therefore regarded as an important target for such putative therapeutics. At a molecular level, a downstream FXR-activated translational repressor of bile acid synthesis has been identified in mice as V-Maf avian musculoaponeurotic fibrosarcoma oncogene homolog G [74]. In addition, the same group has also identified microRNA-144 as a key FXR-mediated regulator of HDL-C metabolism through ATP binding cassette transporter A1 expression modulation [75], thus tying bile signaling neatly into the mammalian lipid metabolism system.

Obeticholic acid (OCA) is a highly potent semi-synthetic derivative of the endogenous FXR-agonist chenodeoxycholic acid, which has demonstrated great promise in the treatment of a number of hepatic diseases [76]. The compound has now progressed through phase II and III clinical studies, effectively reducing several markers tightly associated with the liver failure that is observed in primary biliary cholangitis [77]. The utility of OCA was further strengthened by the successes of the FLINT cohort, a phase IIb NAFLD intervention study, in which the FXR agonist reduced all factors of the NAFLD activity score in concert [78]. However, despite the evident potential, no agonist studies targeting CVD outcomes have been reported in human subjects to date.

### Vascular disease

Beyond lipid metabolism modulation, there is an emerging relationship between gut microbiome status and vascular diseases, such as atherosclerosis, myocardial infarct and stroke [79, 80]. Moreover, there have been compelling reports of associations between the oral microbiome and atherogenesis (reviewed extensively in [81]), while direct sequencing of atherosclerotic plaque microbial DNA itself has revealed a diverse ecosystem dominated predominantly by Proteobacteria [82–84]. Exploration of the gut microbiota in the Bogalusa Heart Study cohort previously demonstrated a link between life-time CVD-risk and gene richness, as well as highlighting several specific diseases associated microbial taxa [85].

Beyond the modification of lipid risk factors, bile signaling has been relatively underexplored as an immunomodulatory pathway directly impacting upon vascular function. Despite this, bile acid receptors have been identified in the vasculature [86] and several bile acid receptor agonists have now demonstrated notable vascular effects in preclinical models, including promotion of reverse cholesterol transport [86, 87]. Two synthetic bile acid receptor ligands have also shown promise in the prevention of atherogenesis. The first of which, WAY-362450, is a potent FXR agonist which was shown to dramatically reduce atherogenesis in two separate transgenic murine models of the disease state [88, 89]. Encouragingly, the FXR-agonist OCA has also shown promise in the prevention of high-fat diet induced atherosclerosis, attenuating plaque formation by 95% and several inflammatory markers associated with CVD progression in the apolipoprotein-E-deficient mouse model [90]. Finally, some intriguing in vitro data are now available supporting a role for FXR-agonists in the prevention of the smooth muscle cell mediated atherogenic effects and migration processes [91], as well as downregulation of the vasoconstrictor endothelin-1 [92].

Preclinical studies with the synthetic TGR5 agonist 6alpha-ethyl-23(S)-methylcholic acid (S-EMCA, INT-777) now also indicate a potential role of TGR5 in the prevention of atherosclerosis. The results demonstrated direct inhibition of NF-κB, which accompanied reduced lipid loading of macrophages and intraplaque inflammation [93]. In terms of natural ligands, taurolithocholic acid (TLCA) has also shown the potential to beneficially modulate vascular function in vitro, significantly reducing tumor necrosis factor-α-induced monocyte infiltration, vascular cell adhesion protein-1 expression and activation of nuclear factor-κB, all through TGR5-mediated pathways [94]. Taken together, these results indicate a potential immunomodulatory role for TGR5 agonists which should be further investigated for use in the clinical context.

### Hypertension and heart failure

The risk of CVD events in patients with concomitant hypertension is estimated to be raised by two to threefold [95]; therefore, elevated blood pressure represents a central modifiable risk factor in prevention of the disease. Hypertension remission is a relatively common phenomenon associated with bariatric surgery and McGavigan and colleagues hypothesized that the alterations observed in microbiome composition and bile metabolism following the procedure may have a role in this effect [96]. In this respect, it is interesting to note that FXR is expressed widely in vascular smooth muscle cells as well as in the heart, particularly the atrial myocardium [97]. In line with this, FXR agonist CDCA has been shown to reduce blood pressure in the spontaneous hypertensive rat model through iNOS expression [98]. Moreover, the FXR agonists PX20606 and OCA have exhibited the potential to partially reduce portal hypertension through modulation of vascular tone [99, 100], a pathway which may have relevance for systemic hypertension.

From a wider CVD risk and heart failure-preserved ejection fraction (HF-PEF) perspective, there exists a clear relationship between hypertension, atrial structural change and atrial fibrillation. Given that altered serum bile acid composition is known to induce atrial arrhythmias in patients with atrial fibrillation [101], it is tempting to speculate that alteration in gut microbiome to enhance a “heart healthy” bile acid profile may have additional beneficial effects on hypertension-induced changes to atrial structure. Nonetheless, despite several intriguing conference abstracts purporting a down-regulation of FXR expression in the left ventricle of spontaneous hypertensive rats with end-stage heart failure [102, 103], formally published confirmatory evidence has yet to surface.

## Caveats of bile acid research

### Animal models

When considering microbiome effects on bile acid signaling it is noteworthy that physiological differences and inconsistencies exist between animal models and the human system. While the gut microbiome can appear relatively similar between rodents and humans at the level of phylum, further probing to the genus level reveals a juxtaposition in the proportions of several bacterial taxa considered of importance to host health and disease [104]. Diet is a powerful regulator of the intestinal microbiome and is therefore an essential consideration when comparing herbivorous rodents to omnivorous humans. Nevertheless, the scientific community now has access to numerous validated and stable murine [105] and porcine [106] models of the ‘humanized’ gut microbiome, as well as animal diets which effectively mimic the macro- and micronutrient composition of our own.

A further consideration, which is highly pertinent to this review, is the differences in serum bile profiles between rodents and humans [107]. Most notably, rodent hepatocytes uniquely express a cytochrome P450 enzyme, CYP2C9, which catalyses the hydroxylation of CDCA and UDCA to α-muricholic acid and β-muricholic acid, respectively [108]. In addition, the proportion of circulating tauro-conjugated bile acids vastly outweighs the glycol-conjugated bile acids in the rodent system, while the reverse is true in the human circulation [107]. These are significant divergences in biochemistry and would undoubtedly impact upon the extrapolation of conclusions drawn from animal models. However, the use of porcine models, which more closely mirror the circulating bile acid profile and general physiology of humans [109, 110], may be sufficient in this regard.

### Tissue specificity

Despite the outlined successes of several FXR mediated therapeutics, significant disparities in the downstream actions of FXR signaling have been observed [111]. Indeed, while some studies report correction of lipid profile [112], an equal number of studies demonstrate the involvement of FXR in dyslipidaemia [113]. However, a number of useful conclusions can be drawn from FXR knock-out mouse models to date. Firstly, much of the reported inconsistency between studies appears to be related to the notably high degree of tissue specificity of FXR. For example, Schmitt et al. previously demonstrated that hepatic FXR expression was protective against hepatic lipid accumulation, while intestinal expression and subsequent FGF15 signaling conferred no such effect [114]. A molecular basis for these tissue specific effects has now been proposed [115]. It has been observed in mice that intestinal FXR-mediated FGF15 signaling reduced hepatic expression of CYP7A1, the rate limiting enzyme of the classic bile acid synthesis pathway, while liver FXR activation primarily repressed hepatic CYP8B1, which is responsible for CA synthesis [115]. This seemingly minute discrepancy likely plays a significant role in the inconsistencies in outcomes observed between murine studies. Indeed, a series of subsequent agonistic experiments have supported the hypothesis that FXR activation within an ileal enterocyte [52, 116] can have vastly contrasting effects to that of hepatocyte stimulation [113, 117]. Therefore, we must acknowledge and continue to examine closely the juxtaposed physiological responses which can be generated in some cases from the same signal, and design such drugs carefully with their desired pharmacokinetics in mind.

## Translational outlook

Over the past several decades, a multiplicity of clinically relevant functions of the microbiome have become evident including inter alia prevention of enteral colonization by pathogens, education of the host immune system, modulation of host metabolism, bile acid deconjugation and caloric salvage, production of essential short chain fatty acids, vitamin B and K synthesis and even participation in cardiac drug metabolism [118, 119]. Associations between gut microbiome profiles and host cardiometabolic health are tantalizing in preclinical models but remain to be demonstrated as causal in man. Thus, we stand on the cusp of translation of this preclinical knowledge to clinical practice [45, 120–122].

Intriguingly, microbiome research is now presenting new insights into the multifactorial mechanisms through which cardiometabolic pathologies arise and in doing so presents exciting prospects for development of novel therapeutics and targets within human subjects. These therapies span the spectrum of prebiotic [123], traditional and next generation probiotics to biotherapeutic compounds whole or in part derived from microbes. Therapeutic strategies can vary from selecting probiotic strains associated with cardiovascular health or alternatively using well described strains as vehicles to deliver molecules or compounds associated with disease mitigation. For instance, seminal studies on the relationship between diet, gut microbiome and TMAO suggest new therapeutic targets for atherosclerosis that are gut microbiome based [7, 8, 124, 125].

Moreover, while statin-based therapies represent the gold standard and have proven highly effective in the management of LDL-C and CVD risk, there remain a subset of at-risk hyperlipidemic patients who are partially or entirely intolerant of this drug class. Therefore, there remains scope for in silico mining and development of novel microbial-derived molecular targets [126], or even the exploitation of whole bacterial therapies as adjuncts to standard management regimes [67, 68, 70, 127]. Indeed, although recent clinical trials indicate that LDLR-promoting proprotein convertase subtilisin/kexin type 9-inhibitor injections are safe and highly potent in circulating LDL-C reduction [128], such antibody-based therapies are inordinately expensive for much of the general population and thus cost-effective adjunctive therapies that are microbiome-based may add incremental benefit in this clinical space.

Already, a number of human studies have indicated promising correlations and direct effects of specific microbial taxa on weight loss, glucose metabolism and correction of other phenotypes of metabolic syndrome [122, 129–132]. Additional future microbiome targets may include regulation of vascular tone, hypertension, diastolic dysfunction, insulin resistance, circadian rhythm, satiety and obesity through alterations in bile salt and short chain fatty acid metabolism [133]. However, it is important to address one caveat within the field of microbiome research: the overwhelming majority of data available are cataloguing of microbial taxa from faeces and their association with host cardiometabolic phenotypes. This essentially represents descriptive phenomenology, which, unfortunately, does not often lead to actionable mechanistic understandings. We now require neatly-designed longitudinal and prospective interventional clinical studies [134], coupled with thoroughly-considered reductionist studies utilizing both germ free and environmentally enriched pre-clinical models with gain/loss of function of specific genetic pathways. These definitive mechanistic studies will act as a prelude to expansion of microbiome therapeutics to the wider clinical community of cardiovascular disease and metabolic syndrome patients.

Although the promise of the personalized medicine predicted upon completion of the Human Genome Project [135] has not yet been fulfilled [136, 137], evidence presented in this review indicates a new and likely equally important host bacterial genome for clinicians to consider, if the design of future personalized therapeutic interventions are to be most effective. Due to the individuality of our intestinal bacteria, some studies have already encountered complex multifactorial and personalized interactions between interventions and the gut microbiome, ultimately stratifying patients into responders and non-responders for some widely prescribed drug classes [138, 139]. As a more integrative understanding of microbiome composition and functionality is obtained, it may even become possible to apply a model not unlike the thrombosis and myocardial infarction (TIMI) score [140], which can broadly assess the likelihood of whether a microbiome composition is contributing to cardiometabolic dysfunction risk by stratifying certain microbial taxa as positively and negatively associated with such a disease state. Indeed, such a tool could make it possible to identify taxa or cohorts of taxa that should be reintroduced to the patient microbiome, in an attempt to rectify functional metabolic imbalances. This has already been elegantly demonstrated with respect to health, frailty and predisposition to disease in the elderly [120].

Finally, we must continue to recognize the power of diet and lifestyle adjustments in augmenting a patient’s disease risk status. In line with this, clinical evidence that implicates diet [141] and exercise [142, 143] as potent factors in beneficially modulating the composition and functionality of our gut microbiome is now emerging. Most recently, a meta-analysis of twenty randomized controlled trials found that intervention with inulin-type fructan fibres confers a reduction of circulating LDL-C in all subjects, while also reducing fasting insulin levels in type-2 diabetic patients [144]. Indeed, dietary ingredients are currently leading candidates in the translational pipeline and perhaps represent the most realistic means of manipulating the gut microbiome for beneficial augmentation of cardiometabolic resilience.

## Conclusions

The gut microbiome represents the physiological ‘system’ which is perhaps most readily modified by external factors, such as diet [141, 145] and pharmacologic applications [146]. Current knowledge implicates reduced bacterial diversity and in turn, reduced gene richness and metabolic capacity within the gut microbiome as a driving factor of metabolic dysfunction and the collapse of host systemic homeostasis. In this review, we have evinced a unity in several of these functional aspects of the microbiome, in the context of cardiometabolic health. In this respect, bile acids have now emerged as one of the central tenets of microbe–host crosstalk, with the potential to impact on many aspects of host cardiometabolic health, including those involved in CVD pathology. While immune interactions are clearly central to the microbiome–host interaction, it is evident that enteric microbes have the potential to produce scores of different bile acid derivatives from deconjugation and dehydoxylation processes. This suggests that bile acids potentially may represent an entire language, rather than a simple signal in this crosstalk. It is clear that we are still a distance away from comprehending the true complexities and subtleties involved in bile pool composition, receptor tissue specificity and nuclear receptor promiscuity, but these pathways may hold the key to many previously unexplained microbiome–host health associations and correlations, and are therefore inherently intriguing in respect of cardiovascular health.
